# The new TNM classification of lymph node metastasis minimises stage migration problems in gastric cancer patients

**DOI:** 10.1038/sj.bjc.6600432

**Published:** 2002-07-02

**Authors:** G de Manzoni, G Verlato, F Roviello, P Morgagni, A Di Leo, L Saragoni, D Marrelli, H Kurihara, F Pasini

**Affiliations:** 1st Division of General Surgery, University of Verona, Verona, Italy; Unit of Epidemiology and Medical Statistics, University of Verona, Verona, Italy; Division of Surgical Oncology, University of Siena, Siena, Italy; Division of Surgery, Forl ì Hospital, Forl ì, Italy; Division of Pathology, Forl ì Hospital, Forl ì, Italy; Division of Surgery, University of Milan, Milan, Italy; Chair of Medical Oncology, University of Verona, Verona, Italy for the Italian Research Group for Gastric Cancer (IRGGC)

**Keywords:** gastric cancer, TNM-UICC classification, lymph node metastasis, stage migration, Will Rogers phenomenon

## Abstract

The present study aimed at investigating whether in gastric cancer patients stage migration occurs with extension of lymphadenectomy, when node metastases are staged according to the new pN classification (UICC 1997). The investigation involved 921 patients, who underwent R0 gastric resection for gastric cancer between 1988 and 1998 in three different Italian centres: Verona (*n*=236), Forlì (*n*=409), Siena (*n*=276). The relation among lymphadenectomy and pN category was assessed by Kendall's partial rank-order correlation coefficient, controlling for depth of tumour invasion. A direct evaluation of the Will Rogers phenomenon was accomplished in the Verona series, by comparing the number of positive nodes actually observed with the number of positive nodes which would have been retrieved by a less extended lymphadenectomy (D1). The number of positive nodes increased remarkably with the enlargement of lymphadenectomy, especially in pT2 patients (from 2.2±3.9 in D1 to 3.9±5.0 in D3) and in pT3/pT4 patients (from 5.1±5.9 in D1 to 11.3±12.6 in D3). Non-parametric statistics highlighted a weak (Kendall's partial T=0.128) but significant (*P*<0.001) correlation between pN category and extension of lymphadenectomy. In the direct analysis of the Verona series, 22 patients out of 230 (9.6%) migrated to a lower pN tier when ignoring positive nodes retrieved from the second and third level. This percentage increased to 39.1% (90 out of 230) when adopting the TNM 87 classification. In conclusion stage migration is of minor importance in gastric cancer patients, staged according to the new pN classification.

*British Journal of Cancer* (2002) **87**, 171–174. doi:10.1038/sj.bjc.6600432 www.bjcancer.com

© 2002 Cancer Research UK

## 

In the past, all staging systems for gastric cancer defined N stage by location of node metastases relative to the primary (Hermanek and Sobin, 1992; JRSGC, 1995). In order to simplify the pN classification, to reduce methodological errors and to achieve a higher reproducibility, the UICC has presented in 1997 a new classification system based on the number of involved nodes (Sobin and Wittekind, 1997).

In the last 3 years many authors compared the new classification with the previous one and showed that both the site and the number of the positive lymph nodes are independent prognostic factors in gastric cancer patients; however the pN categories (especially pN2 and pN3) based on the number are prognostically more homogeneous. Moreover the new TNM system seems to identify a group of high risk patients (pN3 category) with poor long term survival (Hermanek *et al*, 1998; Roder *et al*, 1998; de Manzoni *et al*, 1999; Yoo *et al*, 1999; Katai *et al*, 2000; Klein Kranenbarg *et al*, 2001).

Some authors maintain that the number of positive lymph nodes does not increase further when more than 15 lymph nodes are excised (Hermanek, 1991; Siewert *et al*, 1993). If this statement was correct, another advantage of the new pN classification would be the prevention of stage migration (the so-called Will Rogers phenomenon) (Feinstein *et al*, 1985) which affected the previous pN classification, as shown by Bunt *et al* (1995).

To verify the independence of the new pN classification from the Will Rogers phenomenon, we investigated whether the pN staging is influenced by the surgical procedure adopted, and in particular we addressed the relation between the number of positive nodes and the extension of lymphadenectomy.

## PATIENTS AND METHODS

Between January 1988 and December 1998, 1147 patients underwent gastric resection in three different Italian centres: Verona and Forlì in the north, Siena in central Italy, and, of these, 921 underwent potentially curative resection with complete macroscopic and microscopic removal of the tumour (Verona *n*=236; Forlì *n*=409; Siena *n*=276). Patients were aged 67.2±11.8 years (means±s.d.; range 23–93), 554 were men and 367 were women.

An additional analysis involved the relation between retrieved and metastatic nodes in each of the three tiers. This analysis was feasible in 261 patients from Verona, who underwent gastrectomy between 1988 and 1999 with separate collection of nodes from each tier, as defined by the Japanese Gastric Cancer Association (JGCA, 1998).

Tumours were staged according to both the 1987 and 1997 pathologic classifications (pTNM) of the International Union Against Cancer: the two classifications diverge as lymph node metastases are staged according to the location relative to the primary tumour in pTNM-1987 and according to the number of involved nodes in pTNM-1997 (Hermanek and Sobin, 1992; Sobin and Wittekind, 1997). The histological classification followed the criteria of Lauren. The resection margins were evaluated according to the Western rules assessing the circumferential resection margins (Hermanek, 1995). Lymph node dissection was prospectively classified according to JGCA rules: D1 lymphadenectomy (dissection of all group 1 nodes), D2 lymphadenectomy (dissection of all the group 1 and group 2 nodes) and D3 lymphadenectomy (dissection of all the group 1, group 2 and group 3 nodes) (JGCA, 1998). The demographic and main baseline clinical characteristics of the two cohorts under study are summarised in Table 1

**Table 1:**
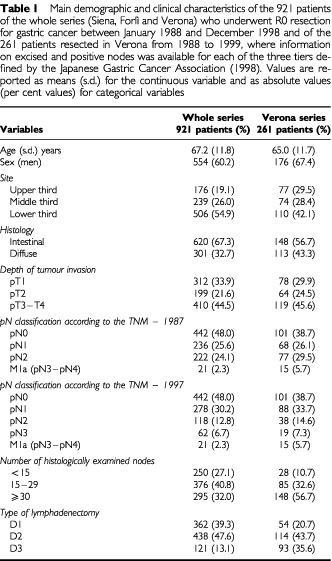
Main demographic and clinical characteristics of the 921 patients of the whole series (Siena, Forlì and Verona) who underwent R0 resection for gastric cancer between January 1988 and December 1998 and of the 261 patients resected in Verona from 1988 to 1999, where information on excised and positive nodes was available for each of the three tiers defined by the Japanese Gastric Cancer Association (1998). Values are reported as means (s.d.) for the continuous variable and as absolute values (per cent values) for categorical variables

.

### Statistical analysis

The relation among the number of positive nodes and the number of excised nodes was evaluated through non-parametric statistics, since the distribution of positive nodes was far from normal, being almost half of the patients N0. First of all, the relation between the two variables was investigated by Kendall's rank-order correlation coefficient T, which ranges from −1 (perfect inverse correlation) to 0 (no correlation at all) and to 1 (perfect direct correlation). As surgeons tend to dissect more lymph nodes in advanced cases, which in turn present higher numbers of metastatic nodes, excised nodes and positive nodes could be related simply because they are both markers of disease progression. To take into account this potential source of bias, the correlation between excised and positive nodes was tested by Kendall's partial rank-order correlation coefficient (Siegel and Castellan, 1988), taking into account also depth of tumour invasion.

To verify whether the extent of lymphadenectomy could elicit a Will Rogers phenomenon, the bi-variate and tri-variate analyses were repeated after replacing the number of excised nodes with type of lymphadenectomy.

To quantify the Will Rogers phenomenon in terms of stage migration, the analyses were repeated after recoding metastatic nodes according to the new and old pN classifications.

The results of the non-parametric statistics were validated in the series from Verona, where information on excised and positive nodes was available for each of the three tiers. Out of 261 cases resected in Verona from 1988 to 1999, 230 had nodes removed in the second and third levels. In these 230 patients the number of positive nodes (and hence the pN97 category) actually observed was compared with the number of positive nodes which would have been retrieved by skipping the second and third levels. As shown in Table 2

**Table 2:**
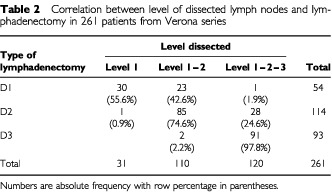
Correlation between level of dissected lymph nodes and lymphadenectomy in 261 patients from Verona series

, a strict relation existed between level of node dissection and extension of lymphadenectomy: D1 were confined to the first (56% of cases) and second (43%) levels, while nearly all D3 (98%) removed nodes from all levels. Thus skipping the second and third levels was nearly equivalent to reducing the extension of lymphadenectomy to D1 and stage migration could be directly evaluated for different extensions of node dissection.

Analyses were performed using the Statistical Product and Service Solutions, SPSS 10.0 for Windows, 2000, SPSS Inc., Chicago IL (Noruris, 1993).

## RESULTS

In the series of 921 patients, the total number of dissected lymph nodes was 23 288, with an average of 25.3±16.3 (means±s.d.) dissected nodes per case (median 21, range 1–108). The mean number of metastatic nodes was 4.3±7.5 (median 1, range 0–74) in the overall series and 8.3±8.7 (median 5, range 1-74) in pN+ patients.

A mean of 13.5±7.5 (median 12, range 1–51) nodes were removed by D1 lymphadenectomy, 28.2±12.4 (median 26, range 7–88) by D2 and 49.8±15.7 (median 47, range 19–108) by D3. Of the 921 patients, 250 (27.1%) had less than 15 nodes examined and therefore they did not completely fulfil the new TNM classification guidelines (Sobin, 2001). These cases were mainly restricted to the subgroup undergoing D1 dissection: indeed, the number of excised lymph nodes was less than 15 in 62.4% (226 out of 362) of D1 lymphadenectomy, but only in 5.5% (24 out of 438) of D2 lymphadenectomy and in none of D3.

Non-parametric analysis revealed a significant correlation between the number of excised nodes and the number of positive nodes (Table 3

**Table 3:**
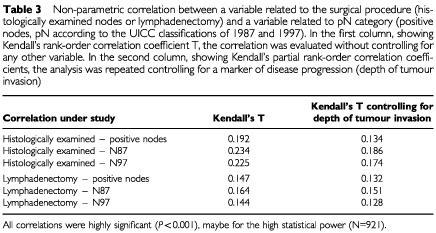
Non-parametric correlation between a variable related to the surgical procedure (histologically examined nodes or lymphadenectomy) and a variable related to pN category (positive nodes, pN according to the UICC classifications of 1987 and 1997). In the first column, showing Kendall's rank-order correlation coefficient T, the correlation was evaluated without controlling for any other variable. In the second column, showing Kendall's partial rank-order correlation coefficients, the analysis was repeated controlling for a marker of disease progression (depth of tumour invasion)

). This correlation (Kendall's T=0.192), although highly significant (*P*<0.001), was rather weak; just for a comparison, the correlation between depth of tumour invasion and positive nodes presented a Kendall's T of 0.525. After controlling for depth of tumour invasion in multivariate analysis, the correlation became even weaker (Kendall's partial T=0.134) but remained statistically significant (*P*<0.001). The number of excised nodes affected to a larger extent the pN category, especially when computed according to the 87 UICC classification. When the number of dissected nodes was replaced by lymphadenectomy, results were similar, with the only exception of the correlation between lymphadenectomy and pN97 category, which was the weakest correlation observed (Table 3).

Table 4

**Table 4:**
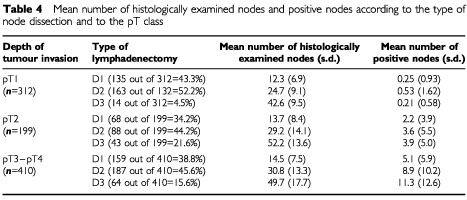
Mean number of histologically examined nodes and positive nodes according to the type of node dissection and to the pT class

shows the mean number of excised and positive nodes as a function of pT class and type of lymphadenectomy. While the number of excised nodes was largely independent from pT class, the number of positive nodes increased remarkably from pT1 to pT3/pT4. As already demonstrated by non-parametric analysis, the number of positive nodes increased remarkably with the enlargement of lymphadenectomy, particularly in pT2 patients (from 2.2±3.9 in D1 to 3.9±5.0 in D3) and even more in pT3/pT4 patients (from 5.1±5.9 in D1 to 11.3±12.6 in D3).

The positive correlation between excised nodes and metastatic nodes observed in the whole series was verified in the 230 patients of the Verona series, with nodes excised beyond the first level: when excluding positive lymph nodes retrieved from the second and third levels, the number of positive nodes decreased slightly from 5.86±8.52 (median 2, range 0–43) to 4.51±6.41 (median 2, range 0–35) and in 22 patients (9.6%) the pN category of TNM '97 was reduced by one level (Table 5

**Table 5:**
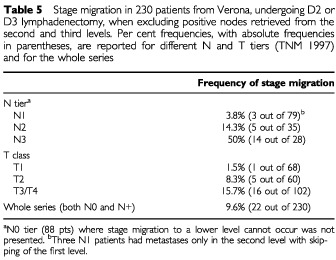
Stage migration in 230 patients from Verona, undergoing D2 or D3 lymphadenectomy, when excluding positive nodes retrieved from the second and third levels. Per cent frequencies, with absolute frequencies in parentheses, are reported for different N and T tiers (TNM 1997) and for the whole series

).

Stage migration was larger when excluding nodes retrieved from the second level: metastatic nodes decreased from 6.88±9.31 to 6.44±8.58 when excluding the third level in 120 patients and from 5.63±8.09 to 4.51±6.41 when ignoring the second level in 230 patients. Accordingly, only 2.5% of patients (3 out of 120) migrated to a lower pN tier when excluding the third level, while 8.3% (19 out of 230) migrated when ignoring the second level.

The Will Rogers phenomenon was particularly pronounced in the pN3 tier, and still present although rare in the pN1 tier. Stage migration increased as a function of depth of tumour invasion (Table 5).

Stage migration was amplified when using the TNM 87 instead of TNM 97. In the 230 patients with nodes excised from the second and third levels, node excision confined to the first level would have resulted in stage migration in the 39.1% of the series (90 out of 230).

## DISCUSSION

The main finding of the present study is that stage migration takes place in some gastric cancer patients in the absence of at least D2 lymphadenectomy. This is suggested by the observation that the number of positive nodes slightly (Kendall's partial T=0.132) but significantly (*P*<0.001) increases with the extension of lymphadenectomy, also when controlling for depth of tumour invasion, and hence for disease progression. This result is further confirmed by the observation that N tier is reduced by one level in 9.6% of Verona patients when skipping the second and third level lymph nodes.

Stage migration increased with the level of nodal involvement (from 3.8% in pN1 tier to 50% in pN3 tier) and with depth of tumour invasion (from 1.5% in the pT1 class to 15.7% in the pT3/pT4 class).

Anyway, the importance of the Will Rogers phenomenon should not be overestimated, as this phenomenon is restricted to a minority of patients and migration involves adjacent pN categories. In this respect the new TNM classification is superior to the old one, which would have allowed stage migration in 40% of patients without dissection of second and third level nodes.

Stage migration was extremely rare when ignoring positive nodes in the third level, with only 2.5% of patients migrating to a lower tier. It was more common when excluding the second level, with 8.3% of patients migrating to a lower level. Hence, the minimum requirement to adequately stage cancer patients is D2 lymphadenectomy, which allows removing lymph nodes beyond the first level in almost all patients (99%). Extension of lymphadenectomy to D3 does not improve staging further, and could be justified only by a favourable impact on survival (de Manzoni *et al*, 1996). The main difference between D2 and D3 resides in the number of nodes excised from the third level (1.1±2.9 in D2 *vs* 9.3±4.8 in D3). However, this large difference has only minor effect on the overall number of positive nodes, since positive nodes retrieved from the third level are quite rare (0.03±0.21 in D2 *vs* 0.54±1.99 in D3). Thus, the extension of lymphadenectomy from D2 to D3 had a limited impact on pN staging.

The authors are not aware of any other studies addressing the Will Rogers phenomenon in gastric cancer after the adoption of the new TNM classification. In an investigation performed with the old pN classification (Bunt *et al*, 1995) the results were similar to those obtained in the present study: 32% of the patients migrated one pN status up, when lymphadenectomy was extended from D1 to D2; moreover, stage migration was more pronounced in advanced TNM stages.

In a recent study (Karpeh *et al*, 2000) stage migration of gastric cancer patients seemed to occur also in the presence of the new TNM classification. Indeed, survival was significantly greater in patients who underwent excision of at least 15 nodes, also after adjusting for pN category. However, evidence for stage migration was only indirect, as better survival with extension of lymphadenectomy could have derived from removal of nodal micrometastasis, as reported for rectal cancer (Tepper *et al*, 2001).

In conclusion, the new pN classification of gastric cancer seems to reduce stage migration elicited by enlargement of lymphadenectomy. Anyway, D2 dissection is necessary to nearly completely avoid the Will Rogers phenomenon; in particular, excision of at least 15 nodes is recommended to achieve an accurate staging in the majority of patients. Further extension of lymphadenectomy to D3 dissection does not modify substantially the pN staging obtained with D2 dissection.
